# Two metal–organic frameworks based on Sr^2+^ and 1,2,4,5-tetra­kis­(4-carb­oxy­phen­yl)benzene linkers

**DOI:** 10.1107/S2056989021011361

**Published:** 2021-11-09

**Authors:** Muhammad Usman, Lydia Ogebule, Raúl Castañeda, Evgenii Oskolkov, Tatiana Timofeeva

**Affiliations:** a Institute of Chemistry, Academia Sinica, Taipei 115, Taiwan; b Department of Chemistry, New Mexico Highlands University, Las Vegas, New Mexico, 87701, USA; c Department of Chemistry, University at Buffalo, Buffalo, New York, 14260, USA

**Keywords:** crystal structure, metal-organic framework, MOF, strontium, 1,2,4,5-tetra­kis­(4-carb­oxy­phen­yl)benzene

## Abstract

Two structurally different metal–organic frameworks based on Sr^2+^ ions and 1,2,4,5-tetra­kis­(4-carb­oxy­phen­yl)benzene linkers have been synthesized solvothermally in different solvent systems and studied with single-crystal X-ray diffraction technique. These are poly[[μ_12_-4,4′,4′′,4′′′-(benzene-1,2,4,5-tetra­yl)tetra­benzoato](di­methyl­formamide)­distrontium(II)], [Sr_2_(C_34_H_18_O_8_)(C_3_H_7_NO)_2_]_*n*_, and poly[tetra­aqua­{μ_2_-4,4′-[4,5-bis­(4-carb­oxy­phen­yl)benzene-1,2-di­yl]dibenzoato}tris­trontium(II)], [Sr_3_(C_34_H_20_O_8_)_2_(H_2_O)_4_]. The differences are noted between the crystal structures and coordination modes of these two MOFs, which are responsible for their semiconductor properties, where structural control over the bandgap is desirable. Hydrogen bonding is present in only one of the compounds, suggesting it has a slightly higher structural stability.

## Abstract 2

Two structurally different metal–organic frameworks based on Sr^2+^ ions and 1,2,4,5-tetra­kis­(4-carb­oxy­phen­yl)benzene linkers have been synthesized solvothermally in different solvent systems, *viz*. poly[[μ_12_-4,4′,4′′,4′′′-(benzene-1,2,4,5-tetra­yl)tetra­benzoato](di­methyl­formamide)­distrontium(II)], [Sr_2_(C_34_H_18_O_8_)(C_3_H_7_NO)_2_]_*n*_, and poly[tetra­aqua­[μ_2_-4,5-bis­(4-carb­oxy­phen­yl)-4,4′-(benzene-1,2-di­yl)dibenzoato]tris­trontium(II)], [Sr_3_(C_34_H_20_O_8_)_2_(H_2_O)_4_]. The differences are noted between the crystal structures and coordination modes of these two MOFs, which are responsible for their semiconductor properties, where structural control over the bandgap is desirable.

## Chemical context

Porous crystalline networks based on metal ion-coordinated organic ligands, known as metal–organic frameworks (MOFs), have been an object of extensive studies for the past two decades. Such inter­est in these materials can be attributed to their fascinating properties and potential applications in a wide range of areas – from luminescent lighting and sensing to gas storage, to semiconductors (Kreno *et al.* 2012 ▸; Zhou *et al.* 2012 ▸; Furukawa *et al.* 2013 ▸; Gassensmith *et al.* 2014 ▸). Their intrinsically unlimited structural and compositional diversity allows the design of structures with virtually any desirable properties. Belonging to the class of coordination compounds, MOFs naturally tend to work particularly well when synth­esized with transition-metal-ion centers, yet they still suffer from several drawbacks, namely the decreased stability, toxicity and relatively high cost of manufacture. In recent years, a new class of alkaline-metal-based MOFs has arisen, providing a solution for the aforementioned problems. Abundant in Earth’s crust and generally non-toxic, ions of Ca, Sr and Ba, for example, have been reported to provide a structurally rich array of compounds with increased stability and unique properties (Kundu *et al.* 2012 ▸). Strontium to date has been a more ‘exotic’ choice in MOF design, with very few structures synthesized and studied. Still, several reports have recently indicated the possibility of Sr–MOF design, which yields structures with unique luminescent (Jia *et al.* 2017 ▸) and semiconducting (Usman *et al.* 2015 ▸) properties, the latter being relatively rare for MOFs and of great inter­est. 
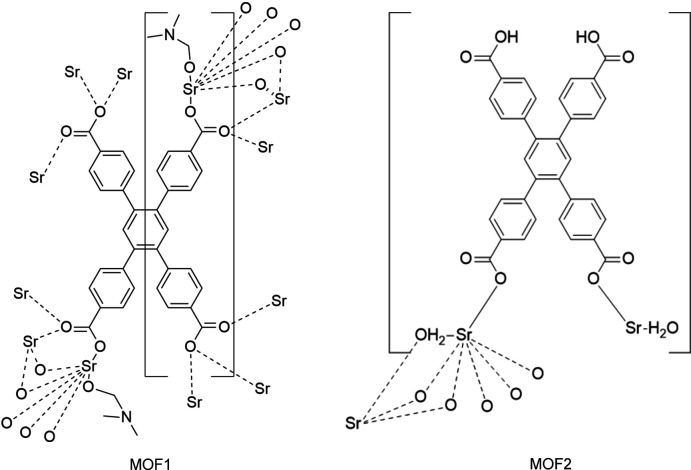


In this work two metal–organic complexes have been synthesized from strontium nitrate as metal ion source and 1,2,4,5-tetra­kis­(4-carb­oxy­phen­yl)benzene as linker under slightly different synthetic conditions (see *Synthesis and crystallization*). For reference purposes these are labeled as **MOF1** and **MOF2**, for the di­methyl­formamide (DMF) and non-DMF containing products, poly[[μ_12_-4,4′,4′′,4′′′-(benzene-1,2,4,5-tetra­yl)tetra­benzoato](di­methyl­formamide)­distron­tium(II)] and poly[tetra­aqua­[μ_2_-4,5-bis­(4-carb­oxy­phen­yl)-4,4′-(benzene-1,2-di­yl)dibenzoato]tris­trontium(II)], respectively.

## Structural commentary

**Figure 1:**
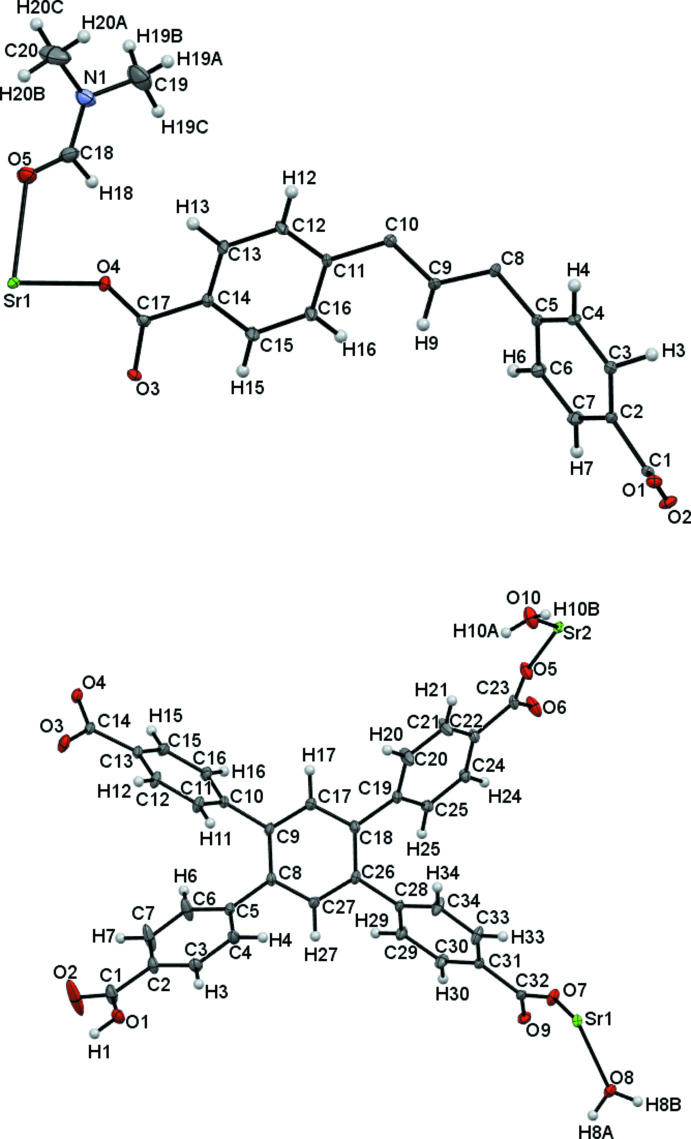
A view of the asymmetric units of **MOF1** (top) and **MOF2** (bottom) with the atom-labeling schemes. Displacement ellipsoids are drawn at the 50% probability level.

Fig. 1 ▸ illustrates the mol­ecular structures of **MOF1** and **MOF2**, specifically their asymmetric units. Selected bond lengths are summarized in Tables 1 ▸ and 2 ▸. In both complexes, an Sr atom with an O_7_ coordination set is present; however, in **MOF2** the asymmetric unit contains two Sr atoms, one seven- and the other eight-coordinated. In **MOF1**, the O_7_ set comprises six O atoms belonging to the carboxyl groups of the ligands (O1–O4) and one atom (O5) belonging to a DMF mol­ecule. In **MOF2**, the seven-coordinated Sr atom is surrounded by five oxygens of carboxyl groups (O4–O7, O9) and two oxygens of water mol­ecules (O8 and O10). The other Sr atom coordinates eight oxygen atoms somewhat similarly: two from water (O8) and six from the carboxyl groups of the ligands (O5–O7). The multidentate nature of the 1,2,4,5-tetra­kis­(4-carb­oxy­phen­yl)benzene ligand, together with the high coordination number of the Sr atom, results in an inter­esting structure for both complexes.

**Figure 2:**
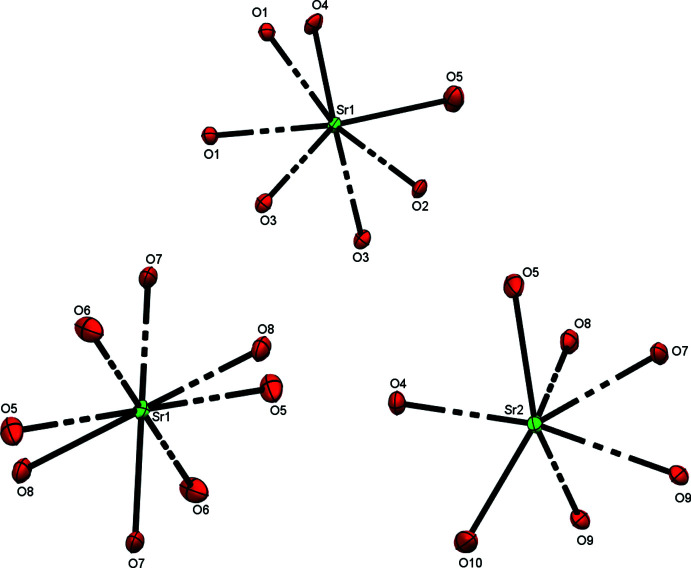
Coordination environments of the Sr atoms in **MOF1** (top) with an O_7_ set and **MOF2** (bottom) with O_8_ and O_7_ sets.

The coordination environments of the Sr ions for both complexes are presented in Fig. 2 ▸. It can be seen that in **MOF1** all available oxygen atoms are coordinated to a metal center, thus all carboxyl groups in the ligands participate in the coordination.

In **MOF2**, atoms O1–O3 are not involved in coordination. While this fact leaves one of the four carboxyl groups (the O1–C1–O2 group) uncoordinated, it does receive some degree of additional stability from hydrogen bonding *via* the O1 atom (see *Supra­molecular features* for more details). The remaining O2 atom shows some degree of disorder due to vibration.

## Supra­molecular features

**Figure 3:**
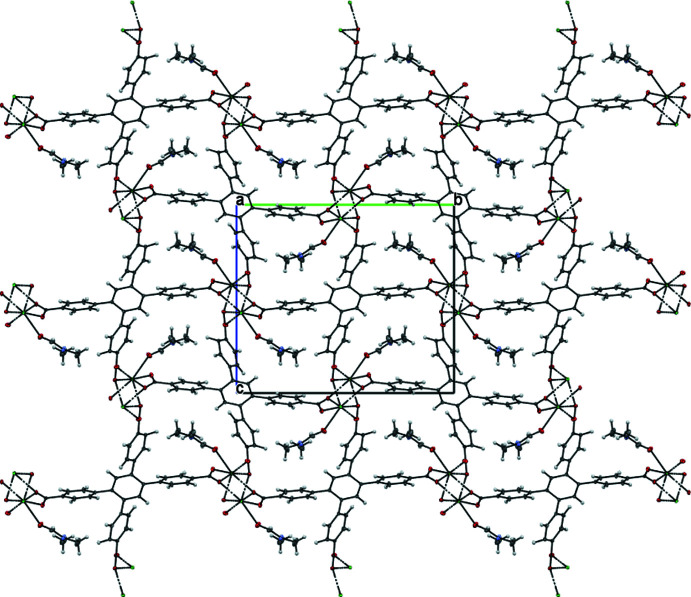
A view along the *a* axis of **MOF1**. Large channel-like pores are occupied by the DMF solvent mol­ecules.

**Figure 4:**
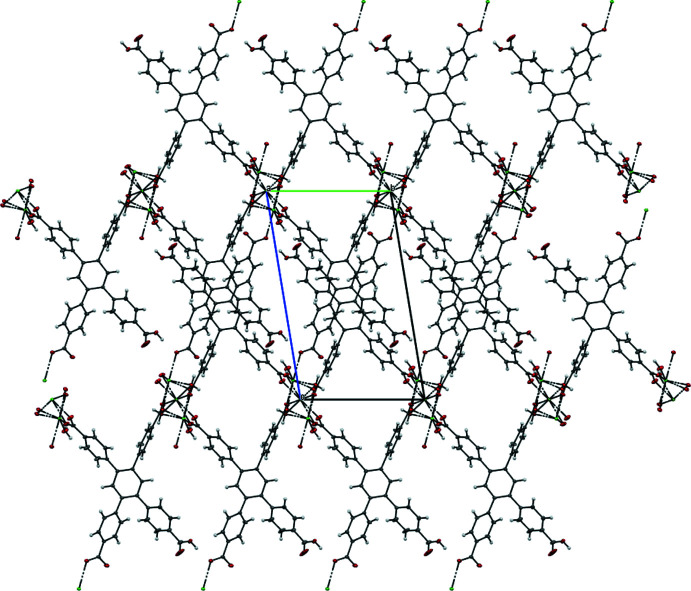
A view along the *a* axis of **MOF2**.

. The packing of **MOF1** is shown in Fig. 3 ▸. While the abundance of carboxyl groups in the ligand provides a lot of potential for hydrogen-bonding sites, only **MOF2** exhibits such inter­actions (Table 3 ▸). Four inequivalent hydrogen bonds of the type O—H⋯O are found in the crystal packing (Fig. 4 ▸), which are likely to contribute to additional structural stability compared to **MOF1**, which is lacking these or any other specific inter­actions. That said, three out of the four hydrogen bonds in **MOF2** stabilize the water mol­ecule rather than the crystal structure directly

## Database survey

No entries were found in the Cambridge Structural Database (CSD version 5.40, update of September 2019; Groom *et al.*, 2016 ▸) for metal–organic frameworks with the same metal–ligand combination as in the title compounds. For MOFs based on the title ligand, shown in the scheme below, and different metal ions, the search yielded eleven matches, among which ions of such metals as Cu, Mg, Zn, Co and Bi were present. The crystal structure of the pure ligand (ZARXOI; Hisaki *et al.* 2017 ▸), shown below, was also found during the search.

The ligand crystallizes in the ortho­rhom­bic system in space group *Pbcn*. MOFs with this linker, however, prefer the triclinic space group *P* 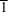 , with some exceptions (see Table 4 ▸). 
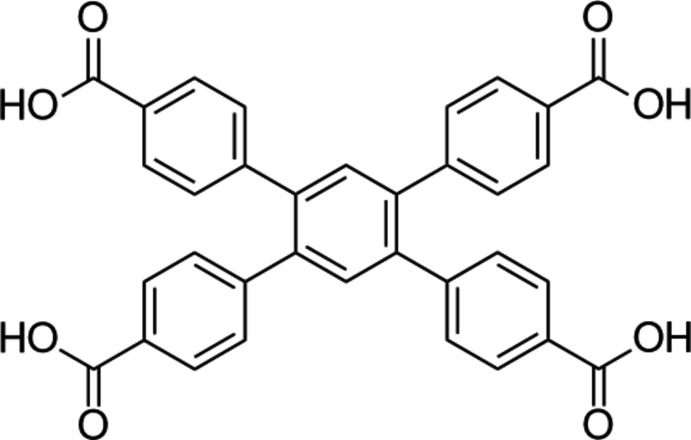


The dihedral angles between the phenyl rings and the central benzene moiety in the ligand are nearly equal: two pairs of 52.66 (18)° and two pairs of 51.05 (18)°. In **MOF1**, the pairwise equality of these angles is conserved; however, both sets of phenyl rings experience significant twists, being 38.08 (11) and 57.88 (11)°, respectively, for each pair. In **MOF2**, an even larger difference is observed, with dihedral angles of 47.44 (8), 60.17 (8), 60.49 (8) and 70.64 (8)° being found between the rings.

## Synthesis and crystallization

**MOF1** was synthesized as follows. Strontium nitrate (0.0212 g, 0.1 mmol), and 1,2,4,5-tetra­kis­(4-carb­oxy­phen­yl) benzene (0.0558 g, 0.1 mmol) were measured, placed in a beaker and dissolved in a mixture of DMF (3 mL) and water (3 mL). The solution was stirred, transferred to a Teflon-lined autoclave and sealed in a reactor, which was placed in the oven at 393 K for 120 h. The autoclave was removed from the oven and allowed to cool to room temperature.

The procedure for **MOF2** differed slightly. The same amounts of the metal precursor and ligand were placed in a beaker and dissolved in a mixture of ethanol (3 mL) and water (3 mL). The solution was stirred, transferred to a Teflon-lined autoclave and sealed in a reactor, which was placed in the oven at 393 K for 120 h. The autoclave was removed from the oven and allowed to cool to room temperature.

After each synthesis, the white crystals of the products were washed with methanol and collected by means of vacuum filtration into a capped vial. An important aspect of this study is the demonstrated possibility of structural control over Sr-based MOFs *via* slight changes in the synthesis conditions. This may be particularly important for semiconducting MOFs, where a structurally tuned bandgap may be desirable.

## Powder X-ray diffraction

**Figure 5:**
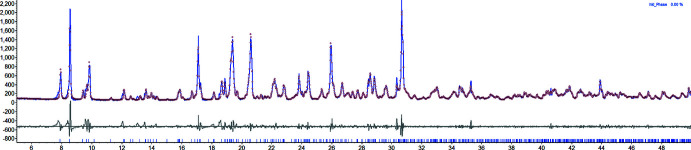
Pawley fit for **MOF2**. The initial parameters were taken from the cif file.

**Figure 6:**
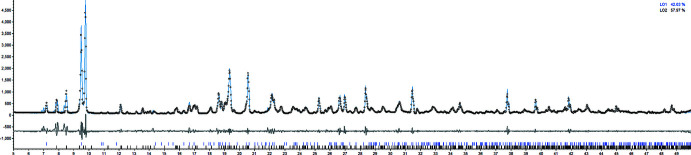
Pawley fit for the bulk solid obtained in the synthesis of **MOF1**. The thick blue lines indicate the crystalline phase for **MOF2** while the thick black lines indicate the crystalline phase for **MOF1**. The initial parameters were taken from the cif files for both MOFs.

In order to identify any potential byproducts or starting materials within the bulk material of **MOF2**, PXRD was conducted using a conventional Bragg–Brentano PXRD instrument. A Pawley fit shows only one crystalline phase (Fig. 5 ▸), and this crystalline phase corresponds to the desired product as it has similar lattice parameters to the single crystal with only a minor increase of 7 Å^3^ of the total unit-cell volume from the single crystal to bulk solid at RT. The resulting lattice parameters for **MOF2** from PXRD are *a* = 9.274 (1), *b* = 11.391 (1), *c* = 19.274 (3) Å, α = 80.38 (1), β = 82.04 (1), γ = 86.11 (1)°, *V* = 1986.3 Å^3^. Unfortunately, in the case of **MOF1**, an analysis by PXRD reveals the phases for **MOF1** and **MOF2** in the same bulk material (Fig. 6 ▸), as in order to do a Pawley fit for this sample both structures are needed. It is possible that for the bulk solid of **MOF1** other additional impurities are present as a few peaks below 10° were not indexed for either **MOF1** or **MOF2** (Fig. 6 ▸).

## Refinement

Crystal data, data collection and structure refinement details are summarized in Table 5 ▸. All C-bound H atoms were positioned geometrically (C—H = 0.95–0.98 Å) and refined using a riding model, *U*_iso_(H) = 1.2*U*_eq_(C). All O-bound H atoms were found from difference Fourier maps and freely refined. For **MOF2**, it was not possible to localize the H atoms at O3 and O6.

## Supplementary Material

Crystal structure: contains datablock(s) global, MOF2, MOF1. DOI: 10.1107/S2056989021011361/yk2158sup1.cif

Structure factors: contains datablock(s) MOF1. DOI: 10.1107/S2056989021011361/yk2158MOF1sup2.hkl

Structure factors: contains datablock(s) MOF2. DOI: 10.1107/S2056989021011361/yk2158MOF2sup3.hkl

CCDC references: 2119605, 2119606

Additional supporting information:  crystallographic information; 3D view; checkCIF report

## Figures and Tables

**Table 1:** Selected bond lengths (Å) for **MOF1** [Chem scheme1]

Sr1—O4	2.5094 (15)	Sr1—O1^iii^	2.5792 (16)
Sr1—O2^i^	2.5170 (16)	Sr1—O3^iv^	2.5848 (16)
Sr1—O3^ii^	2.5180 (15)	Sr1—O1^v^	2.6176 (16)
Sr1—O5	2.5780 (18)		

Symmetry codes: (i) x+2, -y+{\script{1\over 2}}, z+{\script{1\over 2}}; (ii) x+1, y, z; (iii) -x+1, y-{\script{1\over 2}}, -z+{\script{1\over 2}}; (iv) -x+2, -y, -z+1; (v) x+1, -y+{\script{1\over 2}}, z+{\script{1\over 2}}.

**Table 2:** Selected bond lengths (Å) for **MOF2** [Chem scheme1]

Sr1—O7	2.4788 (14)	Sr2—O9^ii^	2.5646 (13)
Sr1—O6^i^	2.5935 (14)	Sr2—O7^iii^	2.6392 (14)
Sr1—O8	2.5938 (14)	Sr2—O4^iv^	2.6598 (14)
Sr1—O5^i^	2.7767 (15)	Sr2—O8^iii^	2.6849 (15)
Sr2—O5	2.5510 (16)	Sr2—O9^iii^	2.8510 (14)

Symmetry codes: (i) x, y-1, z; (ii) x-1, y+1, z; (iii) -x+1, -y+1, -z+2; (iv) -x+1, -y+2, -z+1.

**Table 3:** Hydrogen-bond geometry (Å, °) for **MOF2** [Chem scheme1]

*D*—H⋯*A*	*D*—H	H⋯*A*	*D*⋯*A*	*D*—H⋯*A*
O1—H1⋯O4^i^	0.84	1.79	2.6051 (18)	164
O10—H10*A*⋯O2^v^	0.84 (3)	1.97 (3)	2.795 (2)	168 (3)
O10—H10*B*⋯O6^vi^	0.78 (3)	2.01 (3)	2.787 (2)	173 (3)
O8—H8*B*⋯O3^vii^	0.83 (3)	1.77 (3)	2.5948 (19)	170 (3)

Symmetry codes: (i) x, y-1, z; (v) -x+1, -y+1, -z+1; (vi) x-1, y, z; (vii) x, y-1, z+1.

**Table 4:** Selected CSD data for title ligand-derived MOFs

CSD code	Cation	Coordination number	Space group	Crystal system	Reference
ATIBUD	Zn^2+^	6	*P*\overline{1}	triclinic	(Dissem *et al.* 2021 ▸)
ATICAK	Zn^2+^	6	*P*\overline{1}	triclinic	(Dissem *et al.* 2021 ▸)
ATICEO	Zn^2+^	6	*P*\overline{1}	triclinic	(Dissem *et al.* 2021 ▸)
ATICIS	Cu^2+^	6	*P*\overline{1}	triclinic	(Dissem *et al.* 2021 ▸)
ATICOY	Cu^2+^	6	*P*\overline{1}	triclinic	(Dissem *et al.* 2021 ▸)
ATICUE	Cu^2+^	6	*P*\overline{1}	triclinic	(Dissem *et al.* 2021 ▸)
FIYDEZ	Co^2+^	6	*I*2/*a*	monoclinic	(Dhankhar & Nagaraja 2019 ▸)
MIFKUJ	Zn^2+^	4	*P*\overline{1}	triclinic	(Karra *et al.* 2013 ▸)
MIFLIY	Mg^2+^	6	*C*2/*c*	monoclinic	(Karra, *et al.* 2013 ▸)
MIFMIZ	Ni^2+^	6	*P*\overline{1}	triclinic	(Karra *et al.* 2013 ▸)
MIHMOI	Bi^2+^	5	*C*2/*c*	monoclinic	(Köppen *et al.* 2018 ▸)

**Table 5:** Experimental details

	**MOF1**	**MOF2**
Crystal data		
Chemical formula	[Sr_2_(C_34_H_18_O_8_)(C_3_H_7_NO)_2_]	[Sr_3_(C_34_H_20_O_8_)_2_(H_2_O)_4_]
*M*_r_	875.92	1445.91
Crystal system, space group	Monoclinic, *P*2_1_/*c*	Triclinic, *P*\overline{1}
Temperature (K)	173	100
*a*, *b*, *c* (Å)	5.9350 (2), 18.6130 (8), 16.1256 (7)	9.240 (3), 11.330 (4), 19.414 (7)
α, β, γ (°)	90, 91.853 (2), 90	80.147 (6), 81.815 (7), 85.494 (7)
*V* (Å^3^)	1780.43 (12)	1979.1 (12)
*Z*	2	1
Radiation type	Mo *K*α	Mo *K*α
μ (mm^−1^)	3.06	2.08
Crystal size (mm)	0.41 × 0.12 × 0.10	0.4 × 0.3 × 0.2

Data collection		
Diffractometer	Bruker D8 VENTURE diffractometer	Bruker APEXII CCD
Absorption correction	Multi-scan (*SADABS*; Krause *et al.*, 2015 ▸)	Multi-scan (*SADABS*; Krause *et al.*, 2015 ▸)
*T*_min_, *T*_max_	0.63, 0.75	0.636, 0.746
No. of measured, independent and observed [*I* > 2σ(*I*)] reflections	91152, 5387, 4453	33795, 10986, 9468
*R*_int_	0.092	0.027
(sin θ/λ)_max_ (Å^−1^)	0.713	0.694

Refinement		
*R*[*F* ^2^ > 2σ(*F* ^2^)], *wR*(*F* ^2^), *S*	0.033, 0.113, 0.87	0.030, 0.085, 1.08
No. of reflections	5387	10986
No. of parameters	246	429
H-atom treatment	H-atom parameters constrained	H atoms treated by a mixture of independent and constrained refinement
Δρ_max_, Δρ_min_ (e Å^−3^)	0.94, −0.79	0.58, −0.40

Computer programs: *APEX3* and *SAINT* (Bruker, 2017 ▸), *SHELXT2014/5* (Sheldrick, 2015*a* ▸), *SHELXL2018/3* (Sheldrick, 2015*b* ▸), *Mercury* (Macrae *et al.*, 2020 ▸) and *OLEX2* (Dolomanov *et al.*, 2009 ▸).

## supplementary crystallographic information

### Poly[[µ12-4,4',4'',4'''-(benzene-1,2,4,5-tetrayl)tetrabenzoato](dimethylformamide)distrontium(II)] (MOF1) Crystal data

**Table:**

[Sr_2_(C_34_H_18_O_8_)(C_3_H_7_NO)_2_]	*F*(000) = 884
*M**_r_* = 875.92	*D*_x_ = 1.634 Mg m^−^^3^
Monoclinic, *P*2_1_/*c*	Mo *K*α radiation, λ = 0.71073 Å
*a* = 5.9350 (2) Å	Cell parameters from 9912 reflections
*b* = 18.6130 (8) Å	θ = 2.5–30.3°
*c* = 16.1256 (7) Å	µ = 3.06 mm^−^^1^
β = 91.853 (2)°	*T* = 173 K
*V* = 1780.43 (12) Å^3^	Needle, colourless
*Z* = 2	0.41 × 0.12 × 0.10 mm

### Poly[[µ12-4,4',4'',4'''-(benzene-1,2,4,5-tetrayl)tetrabenzoato](dimethylformamide)distrontium(II)] (MOF1) Data collection

**Table:**

Bruker D8 VENTURE diffractometer	5387 independent reflections
Radiation source: microfocus sealed tube, sealed tube	4453 reflections with *I* > 2σ(*I*)
Multilayer mirror monochromator	*R*_int_ = 0.092
Detector resolution: 10.4167 pixels mm^-1^	θ_max_ = 30.4°, θ_min_ = 2.5°
ω and φ scans, narrow frame width, shutterless	*h* = −8→8
Absorption correction: multi-scan (SADABS; Krause *et al.*, 2015)	*k* = −26→26
*T*_min_ = 0.63, *T*_max_ = 0.75	*l* = −22→22
91152 measured reflections	

### Poly[[µ12-4,4',4'',4'''-(benzene-1,2,4,5-tetrayl)tetrabenzoato](dimethylformamide)distrontium(II)] (MOF1) Refinement

**Table:**

Refinement on *F*^2^	Primary atom site location: structure-invariant direct methods
Least-squares matrix: full	Secondary atom site location: difference Fourier map
*R*[*F*^2^ > 2σ(*F*^2^)] = 0.033	Hydrogen site location: inferred from neighbouring sites
*wR*(*F*^2^) = 0.113	H-atom parameters constrained
*S* = 0.87	*w* = 1/[σ^2^(*F*_o_^2^) + (0.1*P*)^2^] where *P* = (*F*_o_^2^ + 2*F*_c_^2^)/3
5387 reflections	(Δ/σ)_max_ = 0.002
246 parameters	Δρ_max_ = 0.94 e Å^−^^3^
0 restraints	Δρ_min_ = −0.79 e Å^−^^3^

### Poly[[µ12-4,4',4'',4'''-(benzene-1,2,4,5-tetrayl)tetrabenzoato](dimethylformamide)distrontium(II)] (MOF1) Special details

**Table:**

Experimental. Crystal suitable for X–ray structure determination was selected under the polarizing microscope, covered with Paratone oil and mounted on a goniometer head using Mitegen cryoloop. Experiment was performed at the low temperature. QUINN software was used to calculate optimal data collection strategy. Data were collected till resolution of 0.71 A and were truncated with XPREP till actual observed resolution.
Geometry. All esds (except the esd in the dihedral angle between two l.s. planes) are estimated using the full covariance matrix. The cell esds are taken into account individually in the estimation of esds in distances, angles and torsion angles; correlations between esds in cell parameters are only used when they are defined by crystal symmetry. An approximate (isotropic) treatment of cell esds is used for estimating esds involving l.s. planes.
Refinement. The systematic absences in the diffraction data were consistent for the stated space group. The position of almost all non—hydrogen atoms were found by direct methods. The remaining atoms were located in an alternating series of least–squares cycles on difference Fourier map.All non–hydrogen atoms were refined in full–matrix anisotropic approximation. All hydrogen atoms were placed in the structure factor calculation at idealized positions and were allowed to ride on the neighboring atoms with relative isotropic displacement coefficients.Final results were tested with CHECKCIF routine and all A–warnings (if any) were addressed on the very top of this file.

### Poly[[µ12-4,4',4'',4'''-(benzene-1,2,4,5-tetrayl)tetrabenzoato](dimethylformamide)distrontium(II)] (MOF1) Fractional atomic coordinates and isotropic or equivalent isotropic displacement parameters (Å^2^)

**Table:**

	*x*	*y*	*z*	*U*_iso_*/*U*_eq_	
Sr1	1.26769 (3)	0.02155 (2)	0.57357 (2)	0.00827 (8)	
O1	−0.0949 (3)	0.55858 (9)	0.06890 (10)	0.0112 (3)	
O2	−0.4151 (3)	0.55104 (9)	0.13843 (10)	0.0136 (3)	
O3	0.6267 (2)	0.07945 (8)	0.52809 (10)	0.0109 (3)	
O4	0.9519 (3)	0.10863 (9)	0.59477 (11)	0.0134 (3)	
O5	1.3861 (3)	0.09879 (10)	0.70033 (12)	0.0221 (4)	
N1	1.3944 (4)	0.20500 (12)	0.76924 (15)	0.0237 (5)	
C1	−0.2063 (3)	0.55326 (11)	0.13488 (13)	0.0087 (4)	
C2	−0.0700 (3)	0.54814 (12)	0.21498 (13)	0.0091 (4)	
C3	0.1403 (3)	0.58190 (12)	0.22287 (13)	0.0100 (4)	
H3	0.132344	0.633313	0.205003	0.012*	
C4	0.2716 (3)	0.57343 (12)	0.29489 (14)	0.0104 (4)	
H4	0.412954	0.597216	0.300185	0.013*	
C5	0.1993 (4)	0.53065 (11)	0.35938 (14)	0.0094 (4)	
C6	−0.0111 (4)	0.49740 (14)	0.35140 (14)	0.0131 (4)	
H6	−0.06246	0.468151	0.395216	0.016*	
C7	−0.1471 (4)	0.50652 (13)	0.27989 (15)	0.0127 (4)	
H7	−0.29139	0.484462	0.275591	0.015*	
C8	0.3496 (4)	0.51761 (11)	0.43368 (14)	0.0094 (4)	
C9	0.4097 (4)	0.44680 (12)	0.45112 (14)	0.0106 (4)	
H9	0.34665	0.409848	0.417002	0.013*	
C10	0.5582 (3)	0.42735 (11)	0.51635 (13)	0.0097 (4)	
C11	0.6141 (4)	0.35008 (11)	0.52796 (13)	0.0096 (4)	
C12	0.8328 (4)	0.32679 (12)	0.54984 (15)	0.0123 (4)	
H12	0.950402	0.361023	0.557342	0.015*	
C13	0.8791 (3)	0.25430 (12)	0.56065 (15)	0.0119 (4)	
H13	1.027204	0.239731	0.577266	0.014*	
C14	0.7129 (3)	0.20260 (11)	0.54761 (14)	0.0097 (4)	
C15	0.4962 (4)	0.22504 (12)	0.52379 (16)	0.0150 (4)	
H15	0.381025	0.190494	0.513177	0.018*	
C16	0.4483 (4)	0.29796 (12)	0.51552 (15)	0.0142 (4)	
H16	0.298689	0.312497	0.501034	0.017*	
C17	0.7695 (3)	0.12412 (12)	0.55842 (13)	0.0089 (4)	
C18	1.2917 (4)	0.15257 (14)	0.72679 (16)	0.0202 (5)	
H18	1.133968	0.156933	0.71609	0.024*	
C19	1.2757 (6)	0.27042 (18)	0.7921 (2)	0.0426 (9)	
H19A	1.343578	0.311797	0.764847	0.064*	
H19B	1.287327	0.276823	0.852445	0.064*	
H19C	1.116626	0.266555	0.774482	0.064*	
C20	1.6365 (5)	0.20386 (19)	0.7840 (2)	0.0397 (8)	
H20A	1.702782	0.247421	0.760936	0.06*	
H20B	1.700094	0.161503	0.757298	0.06*	
H20C	1.670376	0.20188	0.843878	0.06*	

### Poly[[µ12-4,4',4'',4'''-(benzene-1,2,4,5-tetrayl)tetrabenzoato](dimethylformamide)distrontium(II)] (MOF1) Atomic displacement parameters (Å^2^)

**Table:**

	*U*^11^	*U*^22^	*U*^33^	*U*^12^	*U*^13^	*U*^23^
Sr1	0.00658 (11)	0.00830 (11)	0.00978 (12)	0.00054 (6)	−0.00196 (7)	−0.00102 (7)
O1	0.0118 (7)	0.0121 (8)	0.0096 (7)	−0.0008 (6)	0.0000 (6)	−0.0014 (6)
O2	0.0084 (7)	0.0193 (9)	0.0128 (8)	0.0002 (6)	−0.0036 (6)	−0.0017 (6)
O3	0.0109 (7)	0.0073 (7)	0.0142 (8)	−0.0020 (6)	−0.0014 (6)	−0.0011 (6)
O4	0.0104 (7)	0.0106 (8)	0.0188 (8)	0.0039 (6)	−0.0041 (6)	−0.0012 (6)
O5	0.0218 (9)	0.0208 (10)	0.0232 (10)	0.0048 (7)	−0.0053 (7)	−0.0092 (7)
N1	0.0247 (11)	0.0178 (11)	0.0283 (12)	0.0004 (9)	−0.0026 (9)	−0.0089 (9)
C1	0.0104 (9)	0.0064 (10)	0.0092 (9)	−0.0007 (7)	−0.0022 (7)	−0.0014 (7)
C2	0.0094 (9)	0.0091 (10)	0.0088 (9)	0.0023 (8)	−0.0018 (7)	−0.0026 (8)
C3	0.0099 (9)	0.0086 (9)	0.0115 (10)	0.0006 (7)	−0.0021 (8)	−0.0002 (8)
C4	0.0080 (9)	0.0099 (10)	0.0131 (10)	−0.0008 (7)	−0.0038 (7)	−0.0013 (8)
C5	0.0111 (10)	0.0072 (10)	0.0096 (10)	0.0017 (7)	−0.0038 (8)	−0.0007 (7)
C6	0.0143 (10)	0.0132 (10)	0.0118 (11)	−0.0032 (9)	−0.0015 (8)	0.0030 (9)
C7	0.0094 (9)	0.0141 (11)	0.0146 (11)	−0.0023 (8)	−0.0010 (8)	−0.0003 (9)
C8	0.0094 (9)	0.0090 (10)	0.0095 (10)	0.0017 (7)	−0.0030 (8)	0.0020 (7)
C9	0.0134 (9)	0.0083 (10)	0.0098 (10)	0.0002 (8)	−0.0048 (8)	−0.0007 (8)
C10	0.0114 (9)	0.0083 (10)	0.0093 (10)	0.0000 (7)	−0.0017 (7)	0.0001 (7)
C11	0.0121 (9)	0.0053 (9)	0.0112 (10)	0.0017 (7)	−0.0031 (7)	0.0004 (7)
C12	0.0113 (10)	0.0063 (10)	0.0193 (11)	−0.0017 (8)	−0.0015 (8)	0.0009 (8)
C13	0.0071 (9)	0.0090 (10)	0.0195 (11)	0.0022 (7)	−0.0032 (8)	−0.0004 (8)
C14	0.0095 (9)	0.0074 (9)	0.0122 (10)	0.0015 (7)	−0.0011 (8)	0.0001 (8)
C15	0.0110 (10)	0.0090 (10)	0.0246 (12)	−0.0023 (8)	−0.0047 (9)	0.0012 (9)
C16	0.0091 (9)	0.0083 (10)	0.0249 (12)	−0.0004 (7)	−0.0045 (8)	0.0020 (9)
C17	0.0115 (9)	0.0085 (10)	0.0067 (9)	−0.0005 (7)	0.0007 (7)	−0.0009 (7)
C18	0.0186 (11)	0.0243 (13)	0.0172 (12)	0.0013 (10)	−0.0056 (9)	−0.0056 (10)
C19	0.0467 (19)	0.0247 (16)	0.056 (2)	0.0092 (14)	−0.0039 (16)	−0.0189 (16)
C20	0.0273 (15)	0.0388 (19)	0.053 (2)	−0.0104 (13)	−0.0019 (14)	−0.0190 (16)

### Poly[[µ12-4,4',4'',4'''-(benzene-1,2,4,5-tetrayl)tetrabenzoato](dimethylformamide)distrontium(II)] (MOF1) Geometric parameters (Å, º)

**Table:**

Sr1—O4	2.5094 (15)	C5—C8	1.490 (3)
Sr1—O2^i^	2.5170 (16)	C6—C7	1.396 (3)
Sr1—O3^ii^	2.5180 (15)	C6—H6	0.95
Sr1—O5	2.5780 (18)	C7—H7	0.95
Sr1—O1^iii^	2.5792 (16)	C8—C9	1.392 (3)
Sr1—O3^iv^	2.5848 (16)	C8—C10^vii^	1.403 (3)
Sr1—O1^v^	2.6176 (16)	C9—C10	1.398 (3)
Sr1—C3^v^	3.193 (2)	C9—H9	0.95
Sr1—C1^v^	3.318 (2)	C10—C11	1.487 (3)
Sr1—C2^v^	3.346 (2)	C11—C16	1.392 (3)
Sr1—Sr1^vi^	3.7817 (4)	C11—C12	1.402 (3)
Sr1—Sr1^iv^	3.9854 (4)	C12—C13	1.387 (3)
O1—C1	1.274 (3)	C12—H12	0.95
O2—C1	1.243 (2)	C13—C14	1.389 (3)
O3—C17	1.274 (3)	C13—H13	0.95
O4—C17	1.248 (2)	C14—C15	1.394 (3)
O5—C18	1.230 (3)	C14—C17	1.508 (3)
N1—C18	1.329 (3)	C15—C16	1.392 (3)
N1—C20	1.449 (4)	C15—H15	0.95
N1—C19	1.460 (4)	C16—H16	0.95
C1—C2	1.505 (3)	C18—H18	0.95
C2—C7	1.391 (3)	C19—H19A	0.98
C2—C3	1.399 (3)	C19—H19B	0.98
C3—C4	1.387 (3)	C19—H19C	0.98
C3—H3	1.0	C20—H20A	0.98
C4—C5	1.389 (3)	C20—H20B	0.98
C4—H4	0.95	C20—H20C	0.98
C5—C6	1.396 (3)		
			
O4—Sr1—O2^i^	147.34 (5)	C20—N1—C19	117.0 (2)
O4—Sr1—O3^ii^	113.95 (5)	O2—C1—O1	125.8 (2)
O2^i^—Sr1—O3^ii^	73.87 (5)	O2—C1—C2	117.87 (19)
O4—Sr1—O5	73.58 (5)	O1—C1—C2	116.28 (18)
O2^i^—Sr1—O5	77.82 (6)	O2—C1—Sr1^ix^	148.85 (15)
O3^ii^—Sr1—O5	77.34 (6)	O1—C1—Sr1^ix^	46.87 (10)
O4—Sr1—O1^iii^	70.83 (5)	C2—C1—Sr1^ix^	77.98 (11)
O2^i^—Sr1—O1^iii^	141.62 (5)	C7—C2—C3	119.7 (2)
O3^ii^—Sr1—O1^iii^	86.67 (5)	C7—C2—C1	120.01 (19)
O5—Sr1—O1^iii^	130.36 (5)	C3—C2—C1	120.18 (19)
O4—Sr1—O3^iv^	138.90 (5)	C7—C2—Sr1^ix^	121.05 (15)
O2^i^—Sr1—O3^iv^	71.32 (5)	C3—C2—Sr1^ix^	71.58 (12)
O3^ii^—Sr1—O3^iv^	84.36 (5)	C1—C2—Sr1^ix^	75.93 (11)
O5—Sr1—O3^iv^	147.47 (5)	C4—C3—C2	120.0 (2)
O1^iii^—Sr1—O3^iv^	74.15 (5)	C4—C3—Sr1^ix^	115.00 (14)
O4—Sr1—O1^v^	75.82 (5)	C2—C3—Sr1^ix^	83.85 (13)
O2^i^—Sr1—O1^v^	108.02 (5)	C4—C3—H3	111.7
O3^ii^—Sr1—O1^v^	159.75 (5)	C2—C3—H3	111.7
O5—Sr1—O1^v^	122.91 (6)	Sr1^ix^—C3—H3	111.7
O1^iii^—Sr1—O1^v^	79.85 (5)	C3—C4—C5	120.90 (19)
O3^iv^—Sr1—O1^v^	77.49 (5)	C3—C4—H4	119.5
O4—Sr1—C3^v^	95.21 (6)	C5—C4—H4	119.5
O2^i^—Sr1—C3^v^	63.58 (5)	C4—C5—C6	118.8 (2)
O3^ii^—Sr1—C3^v^	134.51 (5)	C4—C5—C8	120.27 (19)
O5—Sr1—C3^v^	78.67 (6)	C6—C5—C8	120.8 (2)
O1^iii^—Sr1—C3^v^	137.38 (5)	C5—C6—C7	120.9 (2)
O3^iv^—Sr1—C3^v^	96.19 (5)	C5—C6—H6	119.5
O1^v^—Sr1—C3^v^	57.55 (5)	C7—C6—H6	119.5
O4—Sr1—C1^v^	65.57 (5)	C2—C7—C6	119.6 (2)
O2^i^—Sr1—C1^v^	106.31 (5)	C2—C7—H7	120.2
O3^ii^—Sr1—C1^v^	179.38 (5)	C6—C7—H7	120.2
O5—Sr1—C1^v^	102.11 (6)	C9—C8—C10^vii^	118.86 (19)
O1^iii^—Sr1—C1^v^	93.50 (5)	C9—C8—C5	117.35 (19)
O3^iv^—Sr1—C1^v^	96.26 (5)	C10^vii^—C8—C5	123.70 (18)
O1^v^—Sr1—C1^v^	20.81 (5)	C8—C9—C10	123.3 (2)
C3^v^—Sr1—C1^v^	45.45 (5)	C8—C9—H9	118.4
O4—Sr1—C2^v^	72.01 (5)	C10—C9—H9	118.4
O2^i^—Sr1—C2^v^	88.00 (5)	C9—C10—C8^vii^	117.9 (2)
O3^ii^—Sr1—C2^v^	153.58 (5)	C9—C10—C11	118.56 (19)
O5—Sr1—C2^v^	80.19 (6)	C8^vii^—C10—C11	123.55 (19)
O1^iii^—Sr1—C2^v^	118.76 (5)	C16—C11—C12	117.72 (19)
O3^iv^—Sr1—C2^v^	108.25 (5)	C16—C11—C10	120.14 (19)
O1^v^—Sr1—C2^v^	44.66 (5)	C12—C11—C10	122.13 (19)
C3^v^—Sr1—C2^v^	24.57 (5)	C13—C12—C11	120.7 (2)
C1^v^—Sr1—C2^v^	26.10 (5)	C13—C12—H12	119.7
O4—Sr1—Sr1^vi^	141.76 (4)	C11—C12—H12	119.7
O2^i^—Sr1—Sr1^vi^	66.18 (4)	C12—C13—C14	121.19 (19)
O3^ii^—Sr1—Sr1^vi^	42.86 (4)	C12—C13—H13	119.4
O5—Sr1—Sr1^vi^	115.48 (4)	C14—C13—H13	119.4
O1^iii^—Sr1—Sr1^vi^	76.98 (3)	C13—C14—C15	118.6 (2)
O3^iv^—Sr1—Sr1^vi^	41.50 (3)	C13—C14—C17	119.93 (18)
O1^v^—Sr1—Sr1^vi^	118.46 (4)	C15—C14—C17	121.48 (19)
C3^v^—Sr1—Sr1^vi^	122.72 (4)	C16—C15—C14	120.1 (2)
C1^v^—Sr1—Sr1^vi^	137.76 (4)	C16—C15—H15	119.9
C2^v^—Sr1—Sr1^vi^	144.14 (4)	C14—C15—H15	119.9
O4—Sr1—Sr1^iv^	68.07 (4)	C11—C16—C15	121.6 (2)
O2^i^—Sr1—Sr1^iv^	135.27 (4)	C11—C16—H16	119.2
O3^ii^—Sr1—Sr1^iv^	125.35 (4)	C15—C16—H16	119.2
O5—Sr1—Sr1^iv^	140.89 (4)	O4—C17—O3	125.9 (2)
O1^iii^—Sr1—Sr1^iv^	40.28 (4)	O4—C17—C14	117.70 (19)
O3^iv^—Sr1—Sr1^iv^	71.39 (3)	O3—C17—C14	116.42 (18)
O1^v^—Sr1—Sr1^iv^	39.57 (3)	O5—C18—N1	124.8 (2)
C3^v^—Sr1—Sr1^iv^	97.11 (4)	O5—C18—H18	117.6
C1^v^—Sr1—Sr1^iv^	54.94 (4)	N1—C18—H18	117.6
C2^v^—Sr1—Sr1^iv^	81.03 (4)	N1—C19—H19A	109.5
Sr1^vi^—Sr1—Sr1^iv^	99.625 (9)	N1—C19—H19B	109.5
C1—O1—Sr1^viii^	121.40 (13)	H19A—C19—H19B	109.5
C1—O1—Sr1^ix^	112.32 (13)	N1—C19—H19C	109.5
Sr1^viii^—O1—Sr1^ix^	100.15 (5)	H19A—C19—H19C	109.5
C1—O2—Sr1^x^	137.05 (15)	H19B—C19—H19C	109.5
C17—O3—Sr1^xi^	136.59 (14)	N1—C20—H20A	109.5
C17—O3—Sr1^iv^	123.11 (13)	N1—C20—H20B	109.5
Sr1^xi^—O3—Sr1^iv^	95.64 (5)	H20A—C20—H20B	109.5
C17—O4—Sr1	136.51 (15)	N1—C20—H20C	109.5
C18—O5—Sr1	127.84 (16)	H20A—C20—H20C	109.5
C18—N1—C20	120.6 (2)	H20B—C20—H20C	109.5
C18—N1—C19	121.7 (2)		

Symmetry codes: (i) *x*+2, −*y*+1/2, *z*+1/2; (ii) *x*+1, *y*, *z*; (iii) −*x*+1, *y*−1/2, −*z*+1/2; (iv) −*x*+2, −*y*, −*z*+1; (v) *x*+1, −*y*+1/2, *z*+1/2; (vi) −*x*+3, −*y*, −*z*+1; (vii) −*x*+1, −*y*+1, −*z*+1; (viii) −*x*+1, *y*+1/2, −*z*+1/2; (ix) *x*−1, −*y*+1/2, *z*−1/2; (x) *x*−2, −*y*+1/2, *z*−1/2; (xi) *x*−1, *y*, *z*.

### Poly[tetraaqua{µ2-4,4'-[4,5-bis(4-carboxyphenyl)benzene-1,2-diyl]dibenzoato}tristrontium(II)] (MOF2) Crystal data

**Table:**

[Sr_3_(C_34_H_20_O_8_)_2_(H_2_O)_4_]	*Z* = 1
*M**_r_* = 1445.91	*F*(000) = 728
Triclinic, *P*1	*D*_x_ = 1.213 Mg m^−^^3^
*a* = 9.240 (3) Å	Mo *K*α radiation, λ = 0.71073 Å
*b* = 11.330 (4) Å	Cell parameters from 6851 reflections
*c* = 19.414 (7) Å	θ = 2.2–29.5°
α = 80.147 (6)°	µ = 2.08 mm^−^^1^
β = 81.815 (7)°	*T* = 100 K
γ = 85.494 (7)°	Block, colourless
*V* = 1979.1 (12) Å^3^	0.4 × 0.3 × 0.2 mm

### Poly[tetraaqua{µ2-4,4'-[4,5-bis(4-carboxyphenyl)benzene-1,2-diyl]dibenzoato}tristrontium(II)] (MOF2) Data collection

**Table:**

Bruker APEXII CCD diffractometer	9468 reflections with *I* > 2σ(*I*)
φ and ω scans	*R*_int_ = 0.027
Absorption correction: multi-scan (SADABS; Krause *et al.*, 2015)	θ_max_ = 29.6°, θ_min_ = 1.1°
*T*_min_ = 0.636, *T*_max_ = 0.746	*h* = −12→12
33795 measured reflections	*k* = −15→15
10986 independent reflections	*l* = −26→26

### Poly[tetraaqua{µ2-4,4'-[4,5-bis(4-carboxyphenyl)benzene-1,2-diyl]dibenzoato}tristrontium(II)] (MOF2) Refinement

**Table:**

Refinement on *F*^2^	0 restraints
Least-squares matrix: full	Hydrogen site location: mixed
*R*[*F*^2^ > 2σ(*F*^2^)] = 0.030	H atoms treated by a mixture of independent and constrained refinement
*wR*(*F*^2^) = 0.085	*w* = 1/[σ^2^(*F*_o_^2^) + (0.0478*P*)^2^ + 0.5586*P*] where *P* = (*F*_o_^2^ + 2*F*_c_^2^)/3
*S* = 1.08	(Δ/σ)_max_ = 0.003
10986 reflections	Δρ_max_ = 0.58 e Å^−^^3^
429 parameters	Δρ_min_ = −0.40 e Å^−^^3^

### Poly[tetraaqua{µ2-4,4'-[4,5-bis(4-carboxyphenyl)benzene-1,2-diyl]dibenzoato}tristrontium(II)] (MOF2) Special details

**Table:**

Geometry. All esds (except the esd in the dihedral angle between two l.s. planes) are estimated using the full covariance matrix. The cell esds are taken into account individually in the estimation of esds in distances, angles and torsion angles; correlations between esds in cell parameters are only used when they are defined by crystal symmetry. An approximate (isotropic) treatment of cell esds is used for estimating esds involving l.s. planes.

### Poly[tetraaqua{µ2-4,4'-[4,5-bis(4-carboxyphenyl)benzene-1,2-diyl]dibenzoato}tristrontium(II)] (MOF2) Fractional atomic coordinates and isotropic or equivalent isotropic displacement parameters (Å^2^)

**Table:**

	*x*	*y*	*z*	*U*_iso_*/*U*_eq_	
Sr1	0.500000	0.000000	1.000000	0.01031 (5)	
Sr2	0.15862 (2)	0.96611 (2)	0.90833 (2)	0.01075 (5)	
O4	0.77813 (13)	0.93490 (11)	0.22606 (6)	0.0152 (2)	
O9	0.96489 (13)	0.12177 (11)	0.95084 (6)	0.0146 (2)	
O7	0.72246 (13)	0.11703 (11)	0.97587 (6)	0.0160 (2)	
O8	0.68307 (14)	−0.14150 (11)	1.06935 (7)	0.0162 (2)	
O1	0.76027 (14)	0.07521 (11)	0.32051 (7)	0.0181 (3)	
H1	0.782266	0.034447	0.287841	0.027*	
O3	0.66572 (15)	0.77992 (12)	0.20373 (6)	0.0200 (3)	
O5	0.43315 (14)	0.91708 (13)	0.88233 (7)	0.0227 (3)	
O6	0.65612 (14)	0.87652 (13)	0.91244 (7)	0.0234 (3)	
O10	−0.06290 (16)	0.93714 (14)	0.85044 (8)	0.0241 (3)	
H10A	−0.043 (3)	0.902 (3)	0.8154 (17)	0.048 (8)*	
H10B	−0.144 (4)	0.926 (3)	0.8668 (17)	0.055 (10)*	
C24	0.72064 (18)	0.70771 (15)	0.81898 (9)	0.0136 (3)	
H24	0.784204	0.706162	0.853560	0.016*	
C32	0.83640 (18)	0.15159 (14)	0.93521 (8)	0.0111 (3)	
C28	0.77431 (18)	0.37379 (14)	0.73685 (8)	0.0113 (3)	
C8	0.76849 (18)	0.44315 (15)	0.53917 (8)	0.0119 (3)	
C22	0.59333 (18)	0.78304 (15)	0.82027 (9)	0.0141 (3)	
C26	0.75375 (18)	0.44479 (15)	0.66608 (8)	0.0121 (3)	
C9	0.72867 (18)	0.56715 (15)	0.52837 (8)	0.0126 (3)	
C31	0.81624 (18)	0.23133 (14)	0.86662 (8)	0.0111 (3)	
C3	0.7261 (2)	0.20327 (16)	0.43041 (9)	0.0172 (3)	
H3	0.665755	0.138070	0.433079	0.021*	
O2	0.9557 (2)	0.1732 (2)	0.26628 (10)	0.0622 (7)	
C25	0.75471 (18)	0.63506 (15)	0.76738 (9)	0.0136 (3)	
H25	0.840320	0.582839	0.767753	0.016*	
C23	0.55765 (18)	0.86423 (15)	0.87496 (8)	0.0141 (3)	
C27	0.78312 (18)	0.38512 (15)	0.60765 (8)	0.0122 (3)	
H27	0.813999	0.302461	0.614792	0.015*	
C19	0.66454 (19)	0.63775 (15)	0.71480 (8)	0.0137 (3)	
C10	0.72208 (19)	0.63780 (15)	0.45634 (8)	0.0131 (3)	
C2	0.83334 (19)	0.22956 (16)	0.37336 (9)	0.0167 (3)	
C4	0.7072 (2)	0.27333 (15)	0.48400 (9)	0.0178 (3)	
H4	0.633945	0.254908	0.523206	0.021*	
C14	0.71919 (18)	0.83331 (15)	0.24491 (8)	0.0132 (3)	
C5	0.79416 (18)	0.36995 (15)	0.48094 (8)	0.0123 (3)	
C29	0.91564 (19)	0.33494 (16)	0.75236 (9)	0.0173 (3)	
H29	0.997989	0.355855	0.718369	0.021*	
C15	0.81029 (19)	0.80003 (15)	0.36476 (9)	0.0150 (3)	
H15	0.872451	0.864866	0.349027	0.018*	
C13	0.71811 (18)	0.76987 (15)	0.31994 (8)	0.0123 (3)	
C18	0.70443 (18)	0.56721 (15)	0.65602 (8)	0.0134 (3)	
C16	0.8109 (2)	0.73478 (15)	0.43264 (9)	0.0155 (3)	
H16	0.872478	0.756600	0.463100	0.019*	
C30	0.93634 (19)	0.26586 (16)	0.81727 (9)	0.0172 (3)	
H30	1.032707	0.242259	0.827861	0.021*	
C34	0.65490 (19)	0.34253 (17)	0.78723 (9)	0.0185 (4)	
H34	0.558826	0.370510	0.777976	0.022*	
C12	0.62771 (19)	0.67390 (16)	0.34409 (9)	0.0156 (3)	
H12	0.564385	0.653273	0.314045	0.019*	
C17	0.69599 (19)	0.62599 (15)	0.58677 (9)	0.0143 (3)	
H17	0.666923	0.709008	0.579356	0.017*	
C11	0.62918 (19)	0.60830 (16)	0.41139 (9)	0.0162 (3)	
H11	0.567156	0.543338	0.426989	0.019*	
C33	0.67545 (19)	0.27033 (17)	0.85127 (9)	0.0178 (4)	
H33	0.592974	0.247482	0.884745	0.021*	
C21	0.5004 (2)	0.78371 (19)	0.76956 (10)	0.0247 (4)	
H21	0.412135	0.832720	0.770910	0.030*	
C1	0.8559 (2)	0.15670 (18)	0.31465 (10)	0.0227 (4)	
C20	0.5364 (2)	0.71297 (19)	0.71694 (10)	0.0251 (4)	
H20	0.473320	0.715635	0.682029	0.030*	
C6	0.9027 (2)	0.3952 (2)	0.42362 (10)	0.0250 (4)	
H6	0.963271	0.460333	0.420742	0.030*	
C7	0.9219 (2)	0.3245 (2)	0.37053 (11)	0.0321 (5)	
H7	0.996586	0.341418	0.331808	0.039*	
H8A	0.737 (3)	−0.194 (2)	1.0494 (13)	0.032 (7)*	
H8B	0.672 (3)	−0.173 (2)	1.1116 (15)	0.034 (7)*	

### Poly[tetraaqua{µ2-4,4'-[4,5-bis(4-carboxyphenyl)benzene-1,2-diyl]dibenzoato}tristrontium(II)] (MOF2) Atomic displacement parameters (Å^2^)

**Table:**

	*U*^11^	*U*^22^	*U*^33^	*U*^12^	*U*^13^	*U*^23^
Sr1	0.01136 (10)	0.01259 (10)	0.00711 (9)	−0.00104 (7)	−0.00067 (7)	−0.00224 (7)
Sr2	0.01190 (7)	0.01228 (8)	0.00779 (7)	0.00083 (5)	−0.00211 (5)	−0.00080 (5)
O4	0.0191 (6)	0.0167 (6)	0.0094 (5)	−0.0026 (5)	−0.0017 (4)	−0.0003 (5)
O9	0.0140 (6)	0.0154 (6)	0.0135 (6)	0.0022 (5)	−0.0041 (4)	0.0009 (5)
O7	0.0164 (6)	0.0202 (6)	0.0104 (5)	−0.0057 (5)	−0.0031 (5)	0.0032 (5)
O8	0.0196 (6)	0.0158 (6)	0.0113 (6)	0.0006 (5)	−0.0017 (5)	0.0023 (5)
O1	0.0250 (7)	0.0169 (6)	0.0146 (6)	−0.0032 (5)	−0.0023 (5)	−0.0074 (5)
O3	0.0294 (7)	0.0219 (6)	0.0099 (6)	−0.0098 (5)	−0.0066 (5)	0.0007 (5)
O5	0.0202 (6)	0.0334 (8)	0.0147 (6)	0.0106 (6)	−0.0026 (5)	−0.0097 (5)
O6	0.0170 (6)	0.0361 (8)	0.0216 (7)	−0.0007 (6)	−0.0016 (5)	−0.0184 (6)
O10	0.0168 (7)	0.0388 (8)	0.0207 (7)	−0.0069 (6)	−0.0011 (6)	−0.0146 (6)
C24	0.0146 (7)	0.0166 (8)	0.0105 (7)	−0.0002 (6)	−0.0029 (6)	−0.0037 (6)
C32	0.0174 (8)	0.0080 (7)	0.0085 (7)	−0.0005 (6)	−0.0037 (6)	−0.0008 (6)
C28	0.0176 (8)	0.0104 (7)	0.0057 (7)	−0.0017 (6)	−0.0023 (6)	0.0002 (6)
C8	0.0138 (7)	0.0146 (8)	0.0077 (7)	−0.0028 (6)	−0.0004 (6)	−0.0029 (6)
C22	0.0143 (7)	0.0184 (8)	0.0100 (7)	0.0012 (6)	−0.0003 (6)	−0.0054 (6)
C26	0.0144 (7)	0.0148 (8)	0.0068 (7)	−0.0014 (6)	−0.0020 (6)	0.0002 (6)
C9	0.0160 (8)	0.0148 (8)	0.0065 (7)	−0.0011 (6)	−0.0023 (6)	0.0002 (6)
C31	0.0152 (7)	0.0099 (7)	0.0080 (7)	−0.0003 (6)	−0.0026 (6)	−0.0001 (6)
C3	0.0218 (9)	0.0143 (8)	0.0158 (8)	−0.0054 (7)	0.0014 (7)	−0.0040 (6)
O2	0.0733 (14)	0.0800 (14)	0.0415 (11)	−0.0564 (12)	0.0388 (10)	−0.0477 (11)
C25	0.0132 (7)	0.0147 (8)	0.0125 (7)	0.0013 (6)	−0.0018 (6)	−0.0024 (6)
C23	0.0157 (8)	0.0170 (8)	0.0089 (7)	−0.0005 (6)	0.0022 (6)	−0.0035 (6)
C27	0.0160 (7)	0.0109 (7)	0.0093 (7)	−0.0014 (6)	−0.0018 (6)	0.0002 (6)
C19	0.0178 (8)	0.0155 (8)	0.0074 (7)	0.0015 (6)	−0.0016 (6)	−0.0021 (6)
C10	0.0183 (8)	0.0135 (7)	0.0068 (7)	0.0030 (6)	−0.0011 (6)	−0.0014 (6)
C2	0.0191 (8)	0.0215 (9)	0.0112 (8)	−0.0047 (7)	−0.0007 (6)	−0.0073 (7)
C4	0.0248 (9)	0.0141 (8)	0.0132 (8)	−0.0053 (7)	0.0053 (7)	−0.0032 (6)
C14	0.0152 (7)	0.0150 (8)	0.0081 (7)	0.0009 (6)	−0.0016 (6)	0.0008 (6)
C5	0.0154 (7)	0.0135 (7)	0.0079 (7)	0.0010 (6)	−0.0028 (6)	−0.0013 (6)
C29	0.0165 (8)	0.0192 (8)	0.0121 (8)	0.0032 (7)	0.0019 (6)	0.0036 (6)
C15	0.0201 (8)	0.0136 (8)	0.0113 (7)	−0.0024 (6)	−0.0035 (6)	0.0004 (6)
C13	0.0142 (7)	0.0147 (7)	0.0074 (7)	0.0010 (6)	−0.0014 (6)	−0.0009 (6)
C18	0.0161 (7)	0.0157 (8)	0.0091 (7)	0.0017 (6)	−0.0026 (6)	−0.0042 (6)
C16	0.0228 (8)	0.0151 (8)	0.0097 (7)	−0.0015 (7)	−0.0064 (6)	−0.0020 (6)
C30	0.0120 (7)	0.0218 (9)	0.0142 (8)	0.0043 (6)	−0.0007 (6)	0.0036 (7)
C34	0.0137 (8)	0.0282 (9)	0.0122 (8)	−0.0020 (7)	−0.0061 (6)	0.0045 (7)
C12	0.0164 (8)	0.0214 (9)	0.0091 (7)	−0.0042 (7)	−0.0041 (6)	0.0013 (6)
C17	0.0199 (8)	0.0128 (7)	0.0099 (7)	0.0013 (6)	−0.0030 (6)	−0.0010 (6)
C11	0.0183 (8)	0.0200 (8)	0.0094 (7)	−0.0044 (7)	−0.0022 (6)	0.0018 (6)
C33	0.0135 (8)	0.0272 (9)	0.0105 (8)	−0.0048 (7)	−0.0013 (6)	0.0049 (7)
C21	0.0218 (9)	0.0350 (11)	0.0211 (9)	0.0145 (8)	−0.0103 (7)	−0.0165 (8)
C1	0.0270 (10)	0.0293 (10)	0.0146 (8)	−0.0101 (8)	0.0014 (7)	−0.0111 (7)
C20	0.0263 (10)	0.0357 (11)	0.0174 (9)	0.0144 (8)	−0.0133 (7)	−0.0144 (8)
C6	0.0261 (10)	0.0342 (11)	0.0182 (9)	−0.0179 (8)	0.0082 (7)	−0.0162 (8)
C7	0.0312 (11)	0.0487 (13)	0.0207 (10)	−0.0255 (10)	0.0153 (8)	−0.0223 (10)

### Poly[tetraaqua{µ2-4,4'-[4,5-bis(4-carboxyphenyl)benzene-1,2-diyl]dibenzoato}tristrontium(II)] (MOF2) Geometric parameters (Å, º)

**Table:**

Sr1—Sr2^i^	3.9099 (11)	C26—C27	1.401 (2)
Sr1—Sr2^ii^	3.9099 (11)	C26—C18	1.415 (2)
Sr1—O7	2.4788 (14)	C9—C10	1.494 (2)
Sr1—O7^iii^	2.4787 (14)	C9—C17	1.397 (2)
Sr1—O6^i^	2.5935 (14)	C31—C30	1.393 (2)
Sr1—O8	2.5938 (14)	C31—C33	1.397 (2)
Sr1—O8^iii^	2.5938 (13)	C3—H3	0.9500
Sr1—O5^i^	2.7767 (15)	C3—C2	1.385 (2)
Sr1—O5^ii^	2.7767 (15)	C3—C4	1.397 (2)
Sr1—O6^ii^	2.5935 (14)	O2—C1	1.220 (2)
Sr1—C23^i^	3.0535 (18)	C25—H25	0.9500
Sr1—C23^ii^	3.0535 (18)	C25—C19	1.402 (2)
Sr2—Sr2^iv^	4.4117 (11)	C27—H27	0.9500
Sr2—O5	2.5510 (16)	C19—C18	1.491 (2)
Sr2—O9^v^	2.5646 (13)	C19—C20	1.404 (2)
Sr2—O7^ii^	2.6392 (14)	C10—C16	1.398 (3)
Sr2—O4^vi^	2.6598 (14)	C10—C11	1.403 (2)
Sr2—O8^ii^	2.6849 (15)	C2—C1	1.501 (2)
Sr2—O9^ii^	2.8510 (14)	C2—C7	1.391 (3)
Sr2—O10	2.5392 (16)	C4—H4	0.9500
Sr2—C32^ii^	3.1066 (18)	C4—C5	1.396 (3)
O4—C14	1.286 (2)	C14—C13	1.509 (2)
O9—C32	1.273 (2)	C5—C6	1.394 (2)
O7—C32	1.268 (2)	C29—H29	0.9500
O8—H8A	0.85 (3)	C29—C30	1.394 (2)
O8—H8B	0.83 (3)	C15—H15	0.9500
O1—H1	0.8400	C15—C13	1.399 (2)
O1—C1	1.308 (2)	C15—C16	1.397 (2)
O3—C14	1.255 (2)	C13—C12	1.399 (3)
O5—C23	1.256 (2)	C18—C17	1.404 (2)
O6—C23	1.273 (2)	C16—H16	0.9500
O10—H10A	0.84 (3)	C30—H30	0.9500
O10—H10B	0.78 (3)	C34—H34	0.9500
C24—H24	0.9500	C34—C33	1.395 (2)
C24—C22	1.398 (2)	C12—H12	0.9500
C24—C25	1.390 (2)	C12—C11	1.390 (2)
C32—C31	1.502 (2)	C17—H17	0.9500
C28—C26	1.498 (2)	C11—H11	0.9500
C28—C29	1.402 (2)	C33—H33	0.9500
C28—C34	1.391 (2)	C21—H21	0.9500
C8—C9	1.412 (2)	C21—C20	1.392 (2)
C8—C27	1.399 (2)	C20—H20	0.9500
C8—C5	1.495 (2)	C6—H6	0.9500
C22—C23	1.507 (2)	C6—C7	1.394 (3)
C22—C21	1.394 (2)	C7—H7	0.9500
			
Sr2^ii^—Sr1—Sr2^i^	180.0	Sr1—O8—H8B	128.5 (18)
O7—Sr1—Sr2^i^	138.27 (3)	Sr2^ii^—O8—H8A	111.1 (17)
O7—Sr1—Sr2^ii^	41.73 (3)	Sr2^ii^—O8—H8B	93.8 (18)
O7^iii^—Sr1—Sr2^i^	41.73 (3)	H8A—O8—H8B	103 (2)
O7^iii^—Sr1—Sr2^ii^	138.27 (3)	C1—O1—H1	109.5
O7^iii^—Sr1—O7	180.0	Sr2—O5—Sr1^vii^	94.33 (4)
O7^iii^—Sr1—O8^iii^	77.85 (5)	C23—O5—Sr1^vii^	90.30 (10)
O7^iii^—Sr1—O8	102.15 (5)	C23—O5—Sr2	164.35 (13)
O7—Sr1—O8	77.85 (5)	C23—O6—Sr1^vii^	98.52 (10)
O7—Sr1—O8^iii^	102.15 (5)	Sr2—O10—H10A	114 (2)
O7^iii^—Sr1—O5^i^	66.62 (4)	Sr2—O10—H10B	131 (2)
O7^iii^—Sr1—O5^ii^	113.38 (4)	H10A—O10—H10B	108 (3)
O7—Sr1—O5^ii^	66.62 (4)	C22—C24—H24	119.8
O7—Sr1—O5^i^	113.38 (4)	C25—C24—H24	119.8
O7^iii^—Sr1—O6^i^	98.62 (5)	C25—C24—C22	120.34 (15)
O7—Sr1—O6^i^	81.38 (5)	O9—C32—Sr2^ii^	66.58 (9)
O7—Sr1—O6^ii^	98.62 (5)	O9—C32—C31	119.81 (15)
O7^iii^—Sr1—O6^ii^	81.38 (5)	O7—C32—Sr2^ii^	56.97 (8)
O7^iii^—Sr1—C23^i^	81.08 (5)	O7—C32—O9	122.40 (15)
O7—Sr1—C23^i^	98.92 (5)	O7—C32—C31	117.79 (14)
O7—Sr1—C23^ii^	81.08 (5)	C31—C32—Sr2^ii^	167.23 (10)
O7^iii^—Sr1—C23^ii^	98.92 (5)	C29—C28—C26	119.86 (15)
O8^iii^—Sr1—Sr2^i^	43.11 (3)	C34—C28—C26	121.10 (15)
O8^iii^—Sr1—Sr2^ii^	136.89 (3)	C34—C28—C29	119.01 (15)
O8—Sr1—Sr2^ii^	43.11 (3)	C9—C8—C5	123.17 (14)
O8—Sr1—Sr2^i^	136.89 (3)	C27—C8—C9	118.73 (14)
O8—Sr1—O8^iii^	180.0	C27—C8—C5	118.10 (14)
O8^iii^—Sr1—O5^ii^	113.92 (4)	C24—C22—C23	120.60 (15)
O8^iii^—Sr1—O5^i^	66.08 (4)	C21—C22—C24	119.23 (15)
O8—Sr1—O5^i^	113.92 (4)	C21—C22—C23	120.16 (15)
O8—Sr1—O5^ii^	66.08 (4)	C27—C26—C28	117.55 (14)
O8—Sr1—C23^ii^	87.51 (5)	C27—C26—C18	119.17 (14)
O8—Sr1—C23^i^	92.49 (5)	C18—C26—C28	123.26 (14)
O8^iii^—Sr1—C23^ii^	92.49 (5)	C8—C9—C10	122.08 (14)
O8^iii^—Sr1—C23^i^	87.51 (5)	C17—C9—C8	118.99 (14)
O5^ii^—Sr1—Sr2^i^	139.41 (3)	C17—C9—C10	118.92 (15)
O5^i^—Sr1—Sr2^ii^	139.41 (3)	C30—C31—C32	120.86 (14)
O5^ii^—Sr1—Sr2^ii^	40.59 (3)	C30—C31—C33	119.19 (15)
O5^i^—Sr1—Sr2^i^	40.59 (3)	C33—C31—C32	119.94 (15)
O5^ii^—Sr1—O5^i^	180.0	C2—C3—H3	120.2
O5^ii^—Sr1—C23^i^	155.72 (4)	C2—C3—C4	119.53 (17)
O5^i^—Sr1—C23^ii^	155.72 (4)	C4—C3—H3	120.2
O5^ii^—Sr1—C23^ii^	24.28 (4)	C24—C25—H25	119.5
O5^i^—Sr1—C23^i^	24.28 (4)	C24—C25—C19	120.94 (15)
O6^i^—Sr1—Sr2^i^	88.58 (4)	C19—C25—H25	119.5
O6^ii^—Sr1—Sr2^i^	91.42 (4)	O5—C23—Sr1^vii^	65.42 (9)
O6^ii^—Sr1—Sr2^ii^	88.58 (4)	O5—C23—O6	122.20 (15)
O6^i^—Sr1—Sr2^ii^	91.42 (4)	O5—C23—C22	119.59 (15)
O6^i^—Sr1—O8^iii^	107.63 (5)	O6—C23—Sr1^vii^	57.14 (8)
O6^ii^—Sr1—O8	107.63 (5)	O6—C23—C22	118.21 (14)
O6^i^—Sr1—O8	72.37 (5)	C22—C23—Sr1^vii^	172.18 (11)
O6^ii^—Sr1—O8^iii^	72.37 (5)	C8—C27—C26	122.21 (15)
O6^i^—Sr1—O5^i^	48.54 (4)	C8—C27—H27	118.9
O6^i^—Sr1—O5^ii^	131.46 (4)	C26—C27—H27	118.9
O6^ii^—Sr1—O5^i^	131.46 (4)	C25—C19—C18	121.83 (14)
O6^ii^—Sr1—O5^ii^	48.54 (4)	C25—C19—C20	118.21 (15)
O6^ii^—Sr1—O6^i^	180.0	C20—C19—C18	119.91 (15)
O6^i^—Sr1—C23^i^	24.34 (4)	C16—C10—C9	119.67 (15)
O6^i^—Sr1—C23^ii^	155.66 (4)	C16—C10—C11	118.95 (15)
O6^ii^—Sr1—C23^ii^	24.34 (4)	C11—C10—C9	121.38 (16)
O6^ii^—Sr1—C23^i^	155.66 (4)	C3—C2—C1	120.94 (17)
C23^i^—Sr1—Sr2^i^	64.29 (4)	C3—C2—C7	119.59 (16)
C23^ii^—Sr1—Sr2^i^	115.71 (4)	C7—C2—C1	119.46 (16)
C23^i^—Sr1—Sr2^ii^	115.71 (4)	C3—C4—H4	119.4
C23^ii^—Sr1—Sr2^ii^	64.29 (4)	C5—C4—C3	121.16 (16)
C23^ii^—Sr1—C23^i^	180.0	C5—C4—H4	119.4
Sr1^vii^—Sr2—Sr2^iv^	93.96 (3)	O4—C14—C13	119.38 (15)
O4^vi^—Sr2—Sr1^vii^	106.35 (3)	O3—C14—O4	123.88 (15)
O4^vi^—Sr2—Sr2^iv^	137.39 (3)	O3—C14—C13	116.68 (15)
O4^vi^—Sr2—O9^ii^	168.98 (4)	C4—C5—C8	119.14 (15)
O4^vi^—Sr2—O8^ii^	83.07 (4)	C6—C5—C8	121.91 (16)
O4^vi^—Sr2—C32^ii^	166.58 (4)	C6—C5—C4	118.95 (16)
O9^v^—Sr2—Sr1^vii^	105.08 (3)	C28—C29—H29	119.7
O9^ii^—Sr2—Sr1^vii^	82.69 (3)	C30—C29—C28	120.56 (16)
O9^ii^—Sr2—Sr2^iv^	33.35 (3)	C30—C29—H29	119.7
O9^v^—Sr2—Sr2^iv^	37.67 (3)	C13—C15—H15	120.0
O9^v^—Sr2—O4^vi^	100.08 (4)	C16—C15—H15	120.0
O9^v^—Sr2—O9^ii^	71.01 (4)	C16—C15—C13	120.00 (17)
O9^v^—Sr2—O7^ii^	101.82 (4)	C15—C13—C14	121.99 (16)
O9^v^—Sr2—O8^ii^	76.22 (5)	C12—C13—C14	118.85 (15)
O9^ii^—Sr2—C32^ii^	24.19 (4)	C12—C13—C15	119.08 (15)
O9^v^—Sr2—C32^ii^	88.37 (4)	C26—C18—C19	123.52 (14)
O7^ii^—Sr2—Sr1^vii^	38.69 (3)	C17—C18—C26	118.15 (14)
O7^ii^—Sr2—Sr2^iv^	71.57 (4)	C17—C18—C19	118.32 (15)
O7^ii^—Sr2—O4^vi^	142.86 (4)	C10—C16—H16	119.6
O7^ii^—Sr2—O9^ii^	47.66 (4)	C15—C16—C10	120.86 (16)
O7^ii^—Sr2—O8^ii^	73.57 (4)	C15—C16—H16	119.6
O7^ii^—Sr2—C32^ii^	23.75 (4)	C31—C30—C29	120.23 (15)
O8^ii^—Sr2—Sr1^vii^	41.32 (3)	C31—C30—H30	119.9
O8^ii^—Sr2—Sr2^iv^	88.84 (4)	C29—C30—H30	119.9
O8^ii^—Sr2—O9^ii^	100.54 (4)	C28—C34—H34	119.8
O8^ii^—Sr2—C32^ii^	88.92 (4)	C28—C34—C33	120.36 (16)
O5—Sr2—Sr1^vii^	45.08 (3)	C33—C34—H34	119.8
O5—Sr2—Sr2^iv^	137.42 (3)	C13—C12—H12	119.5
O5—Sr2—O4^vi^	76.87 (4)	C11—C12—C13	120.96 (16)
O5—Sr2—O9^ii^	114.15 (4)	C11—C12—H12	119.5
O5—Sr2—O9^v^	144.28 (4)	C9—C17—C18	122.55 (15)
O5—Sr2—O7^ii^	67.83 (4)	C9—C17—H17	118.7
O5—Sr2—O8^ii^	68.06 (5)	C18—C17—H17	118.7
O5—Sr2—C32^ii^	90.17 (4)	C10—C11—H11	119.9
O10—Sr2—Sr1^vii^	178.20 (4)	C12—C11—C10	120.14 (17)
O10—Sr2—Sr2^iv^	85.99 (4)	C12—C11—H11	119.9
O10—Sr2—O4^vi^	74.80 (5)	C31—C33—H33	119.7
O10—Sr2—O9^v^	75.95 (5)	C34—C33—C31	120.58 (16)
O10—Sr2—O9^ii^	96.32 (5)	C34—C33—H33	119.7
O10—Sr2—O7^ii^	139.82 (5)	C22—C21—H21	119.8
O10—Sr2—O8^ii^	140.46 (5)	C20—C21—C22	120.36 (16)
O10—Sr2—O5	134.53 (5)	C20—C21—H21	119.8
O10—Sr2—C32^ii^	117.68 (5)	O1—C1—C2	114.09 (16)
C32^ii^—Sr2—Sr1^vii^	61.05 (3)	O2—C1—O1	123.89 (18)
C32^ii^—Sr2—Sr2^iv^	52.70 (3)	O2—C1—C2	122.01 (18)
C14—O4—Sr2^vi^	122.70 (10)	C19—C20—H20	119.6
Sr2^viii^—O9—Sr2^ii^	108.99 (4)	C21—C20—C19	120.87 (16)
C32—O9—Sr2^viii^	131.25 (11)	C21—C20—H20	119.6
C32—O9—Sr2^ii^	89.23 (10)	C5—C6—H6	120.2
Sr1—O7—Sr2^ii^	99.58 (4)	C5—C6—C7	119.68 (18)
C32—O7—Sr1	149.74 (11)	C7—C6—H6	120.2
C32—O7—Sr2^ii^	99.28 (10)	C2—C7—C6	121.07 (18)
Sr1—O8—Sr2^ii^	95.57 (5)	C2—C7—H7	119.5
Sr1—O8—H8A	120.0 (17)	C6—C7—H7	119.5
			
Sr1—O7—C32—Sr2^ii^	−127.9 (2)	C3—C2—C1—O1	4.7 (3)
Sr1—O7—C32—O9	−114.9 (2)	C3—C2—C1—O2	−175.1 (2)
Sr1—O7—C32—C31	65.6 (3)	C3—C2—C7—C6	−1.2 (3)
Sr1^vii^—O5—C23—O6	−6.63 (18)	C3—C4—C5—C8	179.25 (16)
Sr1^vii^—O5—C23—C22	173.24 (14)	C3—C4—C5—C6	−0.8 (3)
Sr1^vii^—O6—C23—O5	7.2 (2)	C25—C24—C22—C23	−178.49 (16)
Sr1^vii^—O6—C23—C22	−172.70 (13)	C25—C24—C22—C21	0.5 (3)
Sr2^vi^—O4—C14—O3	−11.3 (2)	C25—C19—C18—C26	47.3 (3)
Sr2^vi^—O4—C14—C13	166.13 (10)	C25—C19—C18—C17	−131.15 (18)
Sr2^viii^—O9—C32—Sr2^ii^	114.91 (12)	C25—C19—C20—C21	0.5 (3)
Sr2^ii^—O9—C32—O7	−11.91 (16)	C23—C22—C21—C20	177.01 (19)
Sr2^viii^—O9—C32—O7	102.99 (17)	C27—C8—C9—C10	175.15 (16)
Sr2^viii^—O9—C32—C31	−77.48 (19)	C27—C8—C9—C17	−4.1 (2)
Sr2^ii^—O9—C32—C31	167.61 (13)	C27—C8—C5—C4	59.6 (2)
Sr2^ii^—O7—C32—O9	13.06 (17)	C27—C8—C5—C6	−120.3 (2)
Sr2^ii^—O7—C32—C31	−166.47 (12)	C27—C26—C18—C19	177.53 (16)
Sr2—O5—C23—Sr1^vii^	−107.4 (4)	C27—C26—C18—C17	−4.0 (2)
Sr2—O5—C23—O6	−114.0 (4)	C19—C18—C17—C9	−178.88 (16)
Sr2—O5—C23—C22	65.9 (5)	C10—C9—C17—C18	−177.77 (16)
Sr2^ii^—C32—C31—C30	122.0 (5)	C2—C3—C4—C5	0.3 (3)
Sr2^ii^—C32—C31—C33	−58.9 (6)	C4—C3—C2—C1	−179.59 (17)
O4—C14—C13—C15	−17.0 (2)	C4—C3—C2—C7	0.7 (3)
O4—C14—C13—C12	166.39 (15)	C4—C5—C6—C7	0.3 (3)
O9—C32—C31—C30	5.0 (2)	C14—C13—C12—C11	176.26 (15)
O9—C32—C31—C33	−175.99 (16)	C5—C8—C9—C10	−5.7 (3)
O7—C32—C31—C30	−175.50 (16)	C5—C8—C9—C17	175.09 (16)
O7—C32—C31—C33	3.6 (2)	C5—C8—C27—C26	−176.56 (15)
O3—C14—C13—C15	160.52 (16)	C5—C6—C7—C2	0.7 (4)
O3—C14—C13—C12	−16.0 (2)	C29—C28—C26—C27	69.2 (2)
C24—C22—C23—O5	−169.00 (17)	C29—C28—C26—C18	−111.8 (2)
C24—C22—C23—O6	10.9 (3)	C29—C28—C34—C33	−2.1 (3)
C24—C22—C21—C20	−2.0 (3)	C15—C13—C12—C11	−0.4 (3)
C24—C25—C19—C18	175.55 (16)	C13—C15—C16—C10	1.1 (3)
C24—C25—C19—C20	−2.0 (3)	C13—C12—C11—C10	0.0 (3)
C32—C31—C30—C29	176.78 (16)	C18—C26—C27—C8	1.5 (3)
C32—C31—C33—C34	−178.78 (16)	C18—C19—C20—C21	−177.09 (19)
C28—C26—C27—C8	−179.53 (15)	C16—C10—C11—C12	0.9 (2)
C28—C26—C18—C19	−1.4 (3)	C16—C15—C13—C14	−176.71 (15)
C28—C26—C18—C17	177.02 (16)	C16—C15—C13—C12	−0.2 (2)
C28—C29—C30—C31	2.1 (3)	C30—C31—C33—C34	0.3 (3)
C28—C34—C33—C31	1.9 (3)	C34—C28—C26—C27	−108.97 (19)
C8—C9—C10—C16	−120.06 (19)	C34—C28—C26—C18	70.0 (2)
C8—C9—C10—C11	59.4 (2)	C34—C28—C29—C30	0.1 (3)
C8—C9—C17—C18	1.5 (3)	C17—C9—C10—C16	59.2 (2)
C8—C5—C6—C7	−179.76 (19)	C17—C9—C10—C11	−121.37 (19)
C22—C24—C25—C19	1.5 (3)	C11—C10—C16—C15	−1.5 (2)
C22—C21—C20—C19	1.5 (3)	C33—C31—C30—C29	−2.3 (3)
C26—C28—C29—C30	−178.16 (16)	C21—C22—C23—O5	12.0 (3)
C26—C28—C34—C33	176.15 (17)	C21—C22—C23—O6	−168.11 (18)
C26—C18—C17—C9	2.6 (3)	C1—C2—C7—C6	179.1 (2)
C9—C8—C27—C26	2.7 (3)	C20—C19—C18—C26	−135.20 (19)
C9—C8—C5—C4	−119.61 (19)	C20—C19—C18—C17	46.4 (3)
C9—C8—C5—C6	60.5 (2)	C7—C2—C1—O1	−175.59 (19)
C9—C10—C16—C15	177.99 (15)	C7—C2—C1—O2	4.6 (3)
C9—C10—C11—C12	−178.55 (16)		

Symmetry codes: (i) *x*, *y*−1, *z*; (ii) −*x*+1, −*y*+1, −*z*+2; (iii) −*x*+1, −*y*, −*z*+2; (iv) −*x*, −*y*+2, −*z*+2; (v) *x*−1, *y*+1, *z*; (vi) −*x*+1, −*y*+2, −*z*+1; (vii) *x*, *y*+1, *z*; (viii) *x*+1, *y*−1, *z*.

### Poly[tetraaqua{µ2-4,4'-[4,5-bis(4-carboxyphenyl)benzene-1,2-diyl]dibenzoato}tristrontium(II)] (MOF2) Hydrogen-bond geometry (Å, º)

**Table:**

*D*—H···*A*	*D*—H	H···*A*	*D*···*A*	*D*—H···*A*
O1—H1···O4^i^	0.84	1.79	2.6051 (18)	164
O10—H10*A*···O2^ix^	0.84 (3)	1.97 (3)	2.795 (2)	168 (3)
O10—H10*B*···O6^x^	0.78 (3)	2.01 (3)	2.787 (2)	173 (3)
O8—H8*B*···O3^xi^	0.83 (3)	1.77 (3)	2.5948 (19)	170 (3)

Symmetry codes: (i) *x*, *y*−1, *z*; (ix) −*x*+1, −*y*+1, −*z*+1; (x) *x*−1, *y*, *z*; (xi) *x*, *y*−1, *z*+1.

## References

[bb1] Bruker (2017). *APEX3* and *SAINT*. Bruker AXS Inc. Madison, Wisconsin, USA.

[bb2] Dhankhar, S. S. & Nagaraja, C. M. (2019). *New J. Chem.* **43**, 2163–2170.

[bb3] Dissem, N., Essalhi, M., Ferhi, N., Abidi, A., Maris, T. & Duong, A. (2021). *Dalton Trans.* **50**, 8727–8735. 10.1039/d1dt00426c 34076649

[bb4] Dolomanov, O. V., Bourhis, L. J., Gildea, R. J., Howard, J. A. K. & Puschmann, H. (2009). *J. Appl. Cryst.* **42**, 339–341.

[bb5] Furukawa, H., Cordova, K. E., O’Keeffe, M. & Yaghi, O. M. (2013). *Science*, **341**, 1230444. 10.1126/science.1230444 23990564

[bb6] Gassensmith, J. J., Kim, J. Y., Holcroft, J. M., Farha, O. K., Stoddart, J. F., Hupp, J. T. & Jeong, N. C. (2014). *J. Am. Chem. Soc.* **136**, 8277–8282. 10.1021/ja5006465 24827031

[bb7] Groom, C. R., Bruno, I. J., Lightfoot, M. P. & Ward, S. C. (2016). *Acta Cryst.* B**72**, 171–179. 10.1107/S2052520616003954 PMC4822653 27048719

[bb8] Hisaki, I., Emilya Affendy, N. Q. & Tohnai, N. (2017). *CrystEngComm*, **19**, 4892–4898.

[bb9] Jia, Y. Y., Liu, X. T., Wang, W. H., Zhang, L. Z., Zhang, Y. H. & Bu, X. H. (2017). *Philos. Transact. Ser. A Math. Phys. Eng. Sci.* **375**, 2084. 10.1098/rsta.2016.0026 PMC5179931 27895256

[bb10] Karra, J. R., Huang, Y.-G. & Walton, K. S. (2013). *Cryst. Growth Des.* **13**, 1075–1081.

[bb11] Köppen, M., Meyer, V., Ångström, J., Inge, A. K. & Stock, N. (2018). *Cryst. Growth Des.* **18**, 4060–4067.

[bb12] Krause, L., Herbst-Irmer, R. & Stalke, D. (2015). *J. Appl. Cryst.* **48**, 1907–1913. 10.1107/S1600576714022985 PMC4453166 26089746

[bb13] Kreno, L. E., Leong, K., Farha, O. K., Allendorf, M., Van Duyne, R. P. & Hupp, J. T. (2012). *Chem. Rev.* **112**, 1105–1125. 10.1021/cr200324t 22070233

[bb14] Kundu, T., Sahoo, S. C. & Banerjee, R. (2012). *Chem. Commun.* **48**, 4998–5000. 10.1039/c2cc31135f 22509491

[bb15] Macrae, C. F., Sovago, I., Cottrell, S. J., Galek, P. T. A., McCabe, P., Pidcock, E., Platings, M., Shields, G. P., Stevens, J. S., Towler, M. & Wood, P. A. (2020). *J. Appl. Cryst.* **53**, 226–235. 10.1107/S1600576719014092 PMC6998782 32047413

[bb16] Sheldrick, G. M. (2015*a*). *Acta Cryst.* A**71**, 3–8.

[bb17] Sheldrick, G. M. (2015*b*). *Acta Cryst.* C**71**, 3–8.

[bb18] Usman, M., Mendiratta, S., Batjargal, S., Haider, G., Hayashi, M., Rao Gade, N., Chen, J. W., Chen, Y. F. & Lu, K. L. (2015). *Appl. Mater. Interfaces*, **7**, 22767–22774. 10.1021/acsami.5b07228 26414295

[bb19] Zhou, H. C., Long, J. R. & Yaghi, O. M. (2012). *Chem. Rev.* **112**, 673–674. 10.1021/cr300014x 22280456

